# Supplementary material for: A deep dive into Brazilian health technology assessment: Structure, policies, and processes
Source: PLOS Glob Public Health. 2026 Feb 11;6(2):e0005914. doi: 10.1371/journal.pgph.0005914 (PMC12893538; doi:10.1371/journal.pgph.0005914)
Supplement: S2 Text — (DOCX) [file pgph.0005914.s002.docx]

**A qualitative instrument on:**

**Application of Health Technology Assessment (HTA) In High, Middle, and Low-Income Countries**

**Semi-Structured Interview Guide**

**Length of interview:** 45-60 minutes

**The primary goal of the interview:** To see things the way you see them… more like a conversation with a focus on your understanding, your experience, your opinions, and what you think or feel about the HTA topics covered.

This interview is aimed at evaluating and assessing the HTA understanding, capacity, utilization, and application in the health system. Only national and subnational experts, policymakers, and administrators at public health entities and closely working in HTA fields are invited to participate.

**Confidentiality and anonymity:** All views, perceptions, and perspectives are only used for research purposes and they will be confidentially saved, managed, and shared anonymously. To maintain this and meet the purpose of our research, the interview will be recorded only in voice, and we kindly ask for your personal permission to do so.

**Verbal Consent:** Two individual consents will be secured, the first initial consent before the interview is conducted when the research team communicates with participants via email or phone to invite and explain to them all study information, and the second consent will be obtained when the interview conducts.

**Introduction**

Thanks for participating in this interview ….warm greetings, interviewer introduce himself, and explain briefly the purpose and nature of the interview, as well as its agendas and topics surrounding the HTA.

**Socio-demographic characteristics:**

Age:_____ Gender:_______ Level of education: ___________ Year of experience: ______

Sector affiliation: ______________Location: ___________ Position: ______________

Contact: __________________________ Country : ___________________________

1. **HTA understanding**

As we are interested in the way our interviewees define the HTA concept (rather than starting the interview with our own definition of it), we will ask all interviewees about how they perceive HTA.

1. From your own knowledge and understanding, how do you define the HTA?
2. How do you perceive the HTA in your institution or country?
3. When we mention HTA, what comes to your mind first or what things are emerging to your thinking?
4. What we gain and what we lose from the HTA?

1. **HTA performance and processes**
2. From your perspective, how the HTA is performed, produced, and utilized in the decision-making?
3. What are the strengths and weaknesses of the HTA review process in place?
4. From your perspective, is the political support of HTA is appropriate? Why if not?
5. Do healthcare decisions are made based on HTA evidence in your institution or country?, to what extent HTA plays a role in the decision-making process and for what?
6. How do you evaluate the current health technologies and interventions using HTA?, could you give examples, please?.
7. For the HTA process, what products, devices and tests, procedures undergo HTA and which ones not?, can you elaborate more on this, please?
8. **HTA governance and policy**
   1. **Governance**

- Describe the governance structure of HTA in your country:

1. Is there a clear governing body?, why, if no?
2. Who governs the HTA system (bodies, committees, or agencies)?
3. Who are the actors involved in the HTA system, what are their respective responsibilities?
4. How do you assess their respective roles of HTA actors (producers and users)?
   1. **Policy**

- Based on your knowledge and experience, is there a national policy that manages the work of HTA? Please justify how do you appraise this policy?

1. **HTA capacities and resources**
2. Describe the actual status of the HTA capacities at the institutional and country levels?

- Environmental capacity: legal and political frameworks, norms, culture,
- Organizational capacity: org. structure, policies, and procedures, human resources, physical resources, intellectual resources, networks, leadership
- Individual capacity: skills, experience, and knowledge, attitude and motivations, and education

1. Do you think that there is a need to develop those capacities? how, and what are the priorities to attain this?
2. Explain the reality (status) of financial and human resources allocated to the HTA system? and how the cost of HTA is covered?
3. What further development opportunities in this regard?
4. **Sharing and translating the HTA evidence, knowledge, and outputs**
5. Are you satisfied with the way or the process of HTA evidence or outputs production, dissemination, and utilization?, could you explain why, please?
6. What is required to improve this process among all stakeholders (producers and users)?
7. **HTA and standardization**
8. Does the HTA in your institution or country meet or apply any of the regional and international HTA strategies, standards, models, frameworks, or guidelines such as the WHO, INAHTA, EUnetHTA, ISPOR, International Decision Support Initiative (iDSI)?, explain?
9. What is required to achieve the highest level of HTA quality and best practices?
10. **HTA challenges and obstacles**

- From your perspective, what are the challenges facing the HTA?, these to be listed according to:

1. Institutional level
2. National country level
3. **Opportunities and insights**

- Based on your understanding, what recommendations, solutions, and actions you propose for strengthening a better HTA system?, please list these points according to:

1. Institutional level
2. National country level

**Additional comments, is there anything else you would like to share with us or any questions you have for us?** (This question already starts the debriefing, and the interviewer can even engage in a discussion here).

Interviewer name: ……………………………….

Date: ……………………………………………

**The end …**
