# Supplementary material for: A deep dive into Brazilian health technology assessment: Structure, policies, and processes
Source: PLOS Glob Public Health. 2026 Feb 11;6(2):e0005914. doi: 10.1371/journal.pgph.0005914 (PMC12893538; doi:10.1371/journal.pgph.0005914)
Supplement: Attachment — Submitted filename: Copy of Plos HTA Brazil Paper_Response-Table-2025, 8.11.docx [file pgph.0005914.s004.docx]

Reviewer Comments for Authors from PLOS Global Public Health Decision

A Deep Dive into Brazilian Health Technology Assessment: Structure, Policies, and Processes

Mohammed Alkhaldi*^1,2,3^, Márcia Matos^4,1^, Ali Sweid^5^, Aisha Al Basuoni^7^, Rima Kachach^5,^, Maya Hassan^5^, Patience Mushamiri-Kuzviwanza^7^, Malak Alrubaie^8^, Line Enjalbert^1^, Sara Ahmed^1^

Resubmission to PLOS Global Public Health Decision

We’d like to thank the reviewers for their comments that have substantially improved the quality of our manuscript. Below, we present how we’ve addressed each comment.

| Reviewers’ comments | Description of revisions | Location of revisions |
| --- | --- | --- |
| Section A: Reviewer 1 Comments | | |
| Abstract | | |
| “system analysis research for system thinking and learning” – needs explained | The sentence has been further explained. | Page 2 Line 37 |
| HTA is entirely government-funded- reads as a result but would have thought this would have been known upfront | Good comment. Thank you for the comment. In this study, the **funding source for HTA in Brazil** is primarily government-based, as confirmed by participating stakeholders. While this highlights the strength of the system, we also emphasize the need for **diverse and sustainable funding mechanisms** to enhance efficiency. The study assesses funding in the context of other HTA pillars, including governance, capacities, and community participation, underscoring the importance of a **multidisciplinary staffing team** and active engagement of stakeholders in decision-making processes. | Page 2 line 48 |
| Introduction |  |  |
| World Health Report 2010 – *very old reference to be removed or updated* | We replaced the reference with a more recent one on waste in healthcare has been added.  Olivares-Tirado, P., & Zanga, R. (2023). Waste in health care spending: A scoping review. International Journal of Healthcare Management, 17(2), 330–342. <https://doi.org/10.1080/20479700.2023.2185580> | Page 4 Line 76 |
| “Not only would implementing HTA help in improving the clinical decision-making process, but it is also important to enhance health surveillance and health education and support behavioral changes regarding long-term disease” – how exactly does HTA support this? | Thanks for your comment. The sentences have been revised to clarify how HTA guides evidence-based clinical decision-making but also reinforces health surveillance infrastructure, educational advancement, and behavioral transformation by integrating evidence generation with real-world application. HTA supports a culture of continuous learning and accountability — essential for managing long-term diseases and achieving sustainable health outcomes. A reference has been added. | Page 5 lines 103-105 |
| Research aim | | |
| 1st part of aim is about “the main pillars of the HTA system” – not sure what is meant here by pillars | Pillar refers to components or domains of the HTA system or elements that make up the HTA structure and functions. We further clarified this in the paragraph presenting the study aims. | Page 7 line 144 |
| • 2nd part of aim not well expressed “evaluate the current health technologies, services, and processes in the health system” | We have clarified the second part of the aim as follows: “Evaluate the extent to which and how current health technologies (e.g., digital devices and applications, vaccines, medicines, medical equipment and supplies), and healthcare services and clinical interventions are examined using HTA.” | Page 7 line 152 |
| Objectives seem very wide ranging | Given the purpose of this paper, the objectives were intended to be broad to capture aspects of the system in Brazil that inform the establishment of HTA, and the strengths and areas for improvement needed. The data collection was driven by the aims as stated in the manuscript: The overall aim is linked to comprehensively analyzing the HTA system in Brazil by targeting its pillars/domains for understanding and strengthening purposes. Then the specific objectives come next to explicitly assess the following pillars or domains or components as presented in the manuscript as follows: 1) HTA conceptualization, 2) governance, resources, and capacities, 3) HTA processes related to technologies, drugs, vaccines, equipment, and healthcare services, and then 4) gaps and solutions. | Pages 7 & 8 lines 143-157 |
| Methods |  |  |
| A national HTA system analysis was conducted. This could be better expressed by type of study eg survey, interviews or just research *it is a mixed method approach* | The sentence was modified to highlight that it is a mixed-method approach that includes a survey and an interview. | Page 8 line 160 |
| The survey was expanded by reviewing recent and relevant literature. It also informed the interviews. More could be said on what that literature review involved. | The literature included old and recent peer-reviewed publications produced by relevant international organizations such as WHO, HTAi, ISPOR, institutes of health economics, and networks such as EU-HTA, CADTH, NICE-UK, etc. The relevant content of these publications included tools, manuals, frameworks, surveys, etc., on HTA that were applied and being used in assessing HTA policies, agencies, and processes at the health organizations. Our study tools were developed based on this content, ensuring that our tools are designed to analyze the HTA from a system perspective with a focus on both policy and technical aspects of this system, considering the comprehensiveness, relevance, and robustness of the tools built based on this relevant literature. The authors added a description of what this literature involved. | Page 8 Lines 162-166 |
| Although a protocol is referenced, more detail could be provided. And whilst detail is given in the paragraphs following, the first part of the method’s section leaves the reader wondering who developed the electronic survey etc, why and how were the 13 organisations selected. This section could be better put together. | Thank you for this insightful comment. We have addressed it by incorporating the following paragraph, which includes the requested information.  “This critical and relevant literature included HTA-related surveys, manuals, guidelines, and frameworks. The original version of the survey was developed by the World Health Organization (WHO) and subsequently adapted and advanced by the research team (MA, AAB, and SA), based on a literature review and expert consultations. To gather insights into the technical and operational aspects of HTA, the survey was administered to thirteen organizations representing diverse sectors engaged in HTA-related activities. The selection of these organizations was purposive, guided by predefined criteria detailed in the following sections.” | Page 8 Lines 162-166 |
| “A mixed-method tool used to gather data from thirteen electronic institutional surveys…” reads oddly..mixed methods research was undertaken perhaps edited | Thanks for your comment. The sentence in the first paragraph of the methods section has been edited. | Page 8 line 160 |
| Whilst inclusion criteria given for selecting the thirteen HTA associated organizations, governmental, academic, private, or non-governmental, that operate within the health sector in Brazil, it would be good to know more about their respective roles in HTA or how closely aligned they are eg whilst the sector is noted, are they in service delivery or involved in research? Are they producers or consumers of HTAs -this I think is key to readers’ understanding of the results | In the second part of the study population and sampling section of methods, the authors added an additional descriptive paragraph on these organizations’ roles and involvement in HTA in Brazil. This paragraph reads now as follows:  “These organizations play critical roles within the HTA framework, encompassing healthcare service delivery, regulatory enforcement, research, and evidence generation. Most of them function as both producers and users of HTA evidence, actively contributing to HTA policy formulation, decision-making, production, and use within the Brazilian health system. “  Moreover, the first paragraph of the results section demonstrates further characteristics of these organizations, including the sector involvement, disciplines, scope of work, and role of each organization based on the sector they represented. | Page 11 lines 222-226 |
| Results | | |
| “All thirteen organizations demonstrated a very high understanding of the purpose and concept of HTA”. As they are HTA-related, this seems unsurprising. | Thanks for highlighting this. While it is expected that organizations involved with HTA will have an understanding, the comprehensiveness of this understanding in terms of the conceptualization of the HTA concept, importance, and purpose may have varied, and these questions were an entry point for this comprehensive system analysis. Additionally, the levels of understanding of HTA vary and, in some cases, remain limited. The results in this study and the others we have conducted in other countries will provide data to examine variations in understanding and recognition across countries and stakeholders (policymakers, researchers, healthcare providers, suppliers, etc.) Therefore, this finding remains important as it emphasizes that the sampled institutions possess not only conceptual awareness but also a consistent and comprehensive understanding of HTA’s role and application across different sectors. This alignment and complementarity in the conceptual and practical aspects help to interpret subsequent insights on implementation and system challenges. | No edits occurred |
| Am unclear if the organisations’ are users or providers of HTAs which has implications on how we interpret results. | This comment has been addressed earlier in Comment 5, which pertains to the Methods section. The majority of these organizations serve as both providers and users of HTA evidence, while two academic institutions focus primarily on generating research related to HTA elements. | Page 11 lines 222-226  Page 16 lines 324-326 |
| Experts were from various health sectors (what is meant by various health sectors?) | Thank you for your comment. The experts who participated in the study represent diverse health sectors engaged in HTA activities in Brazil, including the public, private, academic, and NGO sectors. The distribution of experts across these sectors has been clearly described in the Methods section and reiterated in the opening paragraph of the Qualitative Results section.  “This system analysis examined qualitatively the policy perspectives of 9 experts who were interviewed: 4 academicians, 3 from the public sector, and 2 from the private sector.”  Furthermore, the organizations these experts represent have been characterized according to their sectoral affiliation, areas of employment (e.g., multidisciplinary, medical and pharmaceutical sciences, economics, and other health-related fields), and roles within the HTA framework. These clarifications have been incorporated in both the Methods and Results sections, providing detailed information on the organizational type, sector, and primary fields of work of the participating institutions. | Method & Results (give page numbers) |
| Surprised safety is less of a concern than cost-effectiveness? Is safety a first hurdle assessed by other bodies before HTA considered? Needs explained. | In the quantitative results, there are two main findings that assert that safety and cost-effectiveness are important and equally considered. In terms of weight of responses, cost-effectiveness ranked one of the highest dimensions of value for HTA in Brazil, followed by safety. This means that three dimensions, clinical effectiveness, cost and economic evaluation, and safety are also still important dimensions for the Brazilian organizations as well. The study ranked these three highest dimensions based on the weight of responses. The qualitative results supported this that these three dimensions are the most important dimensions in HTA in Brazil.  The authors explained this in the discussion section that safety was less or the third highest dimension for HTA value may be because safety is ensured through regulatory approval processes prior to the HTA assessment, prompting evaluations to focus more on comparative value and economic impact to optimize limited healthcare resources.  In other findings in terms of areas that were frequently covered in HTA processes in Brazil, safety, cost-effectiveness, and cost-economic evaluation were the highest areas.  This means that both are equally considerable for all Brazilian health organizations. This has also been explained in the discussion section.  Below key paragraph that consistently proves our study findings and the interpretation. The first paragraph exists in the results section, and the second paragraph is presented in the interpretation of these findings.  The related results paragraph in the quantitative results reads:  “All organizations unanimously agreed that clinical effectiveness, cost, and economic evaluation are the two most important dimensions of value for HTA, followed by safety (80%), feasibility (68%), organizational impact (56%), equity and equality issues (48%), environmental and political aspects (40%), and ethical issues (24%) (Fig 4). “  “Notably, cost-effectiveness and economic evaluation emerged as the most highly valued dimensions of HTA, followed by safety. This may be because safety is ensured through regulatory approval processes prior to the HTA assessment, prompting evaluations to focus more on comparative value and economic impact to optimize limited healthcare resources. Consistent with these findings, other aligned results confirmed that safety, clinical effectiveness, and economic evaluation are among the most frequently addressed domains within the HTA processes in Brazil.” | Page 18 lines 383-386  Page 26 lines 536-538 |
| Feel some of the results would be known upfront as part of documented processes rather than via research. | . More on contextualization, while some of the results may appear consistent with documented HTA procedures, our findings offer contextualized evidence of how these processes are operationalized within the Brazilian HTA system, highlighting variations, challenges, and practical adaptations not captured in formal documentation. Thus, the research contributes novel empirical data on the functioning and perception of HTA practices among diverse stakeholders. Lastly, this system analysis will be linked with other system analyses that were conducted in different and similar contexts to offer evidence-based learning opportunities for strengthening HTA policies, processes, and experiences.  This comment is similar to the first comment raised about the results. The authors have already addressed this comment and provided this additional response:  The high level of understanding among relevant HTA Brazilian organizations is proven now, which should be assessed to complement it with other pillars or domains of the HTA system, as we seek to comprehensively analyze the system to understand and strengthen it. Assessment of conceptualization on HTA concept, importance, and purpose is an entry point for this comprehensive system analysis, understanding, and strengthening. Additionally, the levels of understanding of HTA remain limited and necessary to be proven to ensure the HTA is cultured, understood, and recognized among all stakeholders, policymakers, researchers, healthcare providers, suppliers, etc. Therefore, this finding remains important as it emphasizes that the sampled institutions possess not only conceptual awareness but also a consistent and comprehensive understanding of HTA’s role and application across different sectors. This alignment and complementarity in the conceptual and practical aspects support the credibility of subsequent insights on implementation and system challenges. | Page 24 lines 518-529 |
| “While medical devices, medicines, vaccines, and clinical interventions are well-established areas for HTA use, equity and equality issues, ethical issues, and patient/citizen/community acceptability”. Sentence’s meaning isn’t expressed very well as the first part refers to different types of technologies, the second part to values (which could be applied to assessments of all types of technologies). Eg, consider changing it to the cost-effectiveness of different technologies is well established but less so equity etc. | Thank you for the comment. As this is in the Results section, we aim to report the findings without interpretation. So, the sentence has been edited, and it reads now as follows:  Cost-effectiveness and economic evaluations of different technologies (medical devices, medicines, vaccines and clinical interventions) are the most important dimensions of value for HTA, which means both are well established in the HTA system in Brazil. However, equity and equality issues, ethical issues, and patient/citizen/community acceptability are less covered in HTA in Brazil, particularly for service delivery models.” | Page 19 lines 395-397 |
| Discussion |  |  |
| States that the findings show a high level of understanding and recognition of the HTA’s importance among Brazilian organizations in public, private, and academic sectors. However, am still unclear how / the extent to which these organisations relate to HTA activities eg is this a biased selection in terms of their closeness to HTA? | This purposive sampling aimed to capture informed perspectives on the Brazilian HTA system rather than a general representation of all health institutions. We have clarified this in the Methods section and now acknowledge in the Discussion that the high level of understanding observed may reflect this targeted sample rather than the broader health sector.  As the study’s objective was to capture in-depth and specific insights from stakeholders directly involved in HTA processes, rather than provide a broader health sector-level perception. It is also important to note that the HTA community in both high-income and low- and middle-income countries tends to be relatively small, with stakeholders, agencies, and processes that are generally well known within the field. To address this, we have further clarified the selection rationale and role of participating organizations in the *Study Population and Sampling* subsection of the *Methods*. | Page 10 lines 218-220  Page 23 lines 513-517 |
| “Coordinated efforts are required to improve HTA efficacy..” what is envisaged by efficacy here? | Thank you for this comment. The intention was to refer to the effectiveness and efficiency of HTA processes, that is, how well HTA functions are coordinated, implemented, and translated into evidence-informed policy decisions. The authors have revised the sentence in the abstract part and it reads now as follows:  “Coordinated efforts are required to improve effectiveness and efficiency of HTA processes in informing healthcare decision-making to address issues such as inadequate impact measurement. “ | Page 3 Lines 54-56 |
| Figure 1 – unclear how to interpret this as total % > 100%. Sure its fine but reader needs help to interpret. | Thank you for the comment. The percentages in Figure 1 exceed 100% because multiple stakeholders may receive the same HTA report. Meaning percentages exceed 100% because responding organizations could select multiple answers. Each percentage represents the proportion of a responding organization that selected that option. This also applies to many questions in the study survey. We have clarified this in the figure caption and text to help readers interpret the results correctly. | Page 17 line 344 |
| Figure 2 – title not well explained nor are the numbers on the figure, | Thank you for your observation. Fig 2 has been revised by editing the title and accompanying description to better explain its content and clarify what the numbers represent. | Page 17 line 355 |
| Figure 3 - same goes for Fig 3 regarding the numbers eg is this per organisation? | Thank you for this comment. Fig 3 has been revised by editing the results section and the title. Meaning percentages exceed 100% because responding organizations could select multiple answers. Each percentage represents the proportion of a responding organization that selected that option. | Page 18 line 374 |
| Figure 4 – some explanation needed re: safety being less of a consideration than cost-effectiveness | The explanation regarding why safety ranked as a lower consideration compared to cost-effectiveness and economic evaluations has been included in the Discussion section as stated below: “Notably, cost-effectiveness and economic evaluation emerged as the most highly valued dimensions of HTA, followed by safety. This may be because safety is ensured through regulatory approval processes prior to the HTA assessment, prompting evaluations to focus more on comparative value and economic impact to optimize limited healthcare resources. “However, the authors have made some additional results descriptions for more clarity. | Page 19 lines 395-397  Page 24 lines 518-521 |
| title is “A Deep Look….” . More colloquial to say “A Deep Dive…” | Thanks for your comment. This has been edited. | The Title |
| “system analysis study” – doesn’t seem quite right in terminology – just say a study which you then analyse using its data. | Thanks for your comment. The terminologies have been edited to a mixed-method study. | Page 8 line 160 |
| Overall edit needed as sometimes goes into a lot of detail perhaps unnecessarily eg describing qualitative research, lots of detail on software used etc, but at other times the detail is lacking, especially a lit review mentioned twice to inform both the survey and interviews but not discussed. And whether the 13 organisations produce or consume HTAs. | Thank you for your feedback. We have revised the manuscript to reduce excessive methodological details while retaining essential information, clarify how the literature review informed both the survey and interviews, and specify, in both the methods and results sections, whether the 13 organizations primarily regulate, produce, consume, or both produce and use HTA. Additionally, the authors will collaborate with the journal production team on any further edits needed to enhance clarity, balance, and overall readability of the final version of the manuscript. | Methods & Results section |
| Section B: Reviewer 2 Comments | | |
| The manuscript references a protocol, but additional methodological detail is needed to help readers understand the methods. | We appreciate the reviewer’s valuable feedback. While the cited protocol provides a general overview of the study methods, the Methods section of this manuscript has now been expanded to include more detailed descriptions specific to the study conducted. In particular, we have elaborated on the tool development process, the selection of organizations and experts, and the study population and sampling strategy for both the quantitative and qualitative components. These additions will enable readers to fully understand the methods without needing to consult the external protocol. The revised content can be found in the relevant subsections of the Methods section of the revised manuscript. | Method Section |
| To clarify the methods, include a flowchart summarizing invitations, eligibility, consent, and completions (survey and interviews). If a figure isn’t feasible, provide the same counts in the Methods text. | Thank you for making this important comment. The authors have provided this necessary methodological information in separate subsections of the Methods section in the revised manuscript. Specifically, twenty eligible organizations were invited to participate, of which thirteen consented and completed one organizational survey. Regarding the experts, twelve were invited, and nine accepted the invitation and participated in individual interviews. | Method Section |
| The Introduction states that CONITEC was created in 2011 under Law 8,080, whereas the Discussion says “CONITEC, established in 2012 by the Ministry of Health.” Please verify the correct year and use it consistently throughout. Also, your data collection window is 3 May 2021–22 April 2022, yet the Discussion includes developments after that period (e.g., Law 14,313 of 2022 and a 2025 article on CONITEC). | We thank the reviewer for this helpful observation. We have verified that CONITEC was officially established in 2011 under Law No. 12,401 of April 28, 2011, which amended Law No. 8,080 of 1990, and was regulated by Decree No. 7,646 of December 21, 2011. All references have been corrected for consistency.  Regarding the timeline, we have removed and reframed post-study developments (e.g., Law 14,313 of 2022 and later publications) to clarify that they were not part of the analyzed data but are mentioned only for contextual background. | Page 26 line 546 |
| If a figure or table underpins your main claims, include it in the main manuscript (not just the supplement). For this paper, that means keeping in the main text: the HTA pathway schematic, a bar chart of the top impediments, a timeline of key laws/CONITEC milestones, a table showing who uses HTA outputs, and a sample-characteristics table (N, sectors). | We appreciate this helpful suggestion. The authors have consulted the *PLOS Global Public Health* journal’s guidelines. A limited number of key figures should appear in the main text, while additional figures may be included as supplementary materials would be a useful way. The authors will carefully consider this suggestion during the production process to ensure alignment with journal requirements and to maintain the relevance and consistency of the manuscript and its figures. Thank you. |  |
