# Supplementary material for: A deep dive into Brazilian health technology assessment: Structure, policies, and processes
Source: PLOS Glob Public Health. 2026 Feb 11;6(2):e0005914. doi: 10.1371/journal.pgph.0005914 (PMC12893538; doi:10.1371/journal.pgph.0005914)
Supplement: Attachment — Submitted filename: Plos HTA Brazil Paper_Response-Table-2026.docx [file pgph.0005914.s005.docx]

Reviewer Comments for Authors from PLOS Global Public Health Decision

A Deep Dive into Brazilian Health Technology Assessment: Structure, Policies, and Processes

Mohammed Alkhaldi*^1,2,3^, Márcia Matos^4,1^, Ali Sweid^5^, Aisha Al Basuoni^7^, Rima Kachach^5,^, Maya Hassan^5^, Patience Mushamiri-Kuzviwanza^7^, Line Enjalbert^1^, Sara Ahmed^1^

Resubmission to PLOS Global Public Health Decision

We’d like to thank the reviewers for their comments that have substantially improved the quality of our manuscript: Below we present how we’ve addressed each comment.

| Reviewers’ comment | Description of revisions | Location of revisions |
| --- | --- | --- |
| Abstract, line 48: The sentence “HTA is entirely government-funded that requiring diverse and 49 sustainable sources…” needs editing. | The sentence has been revised again and it was edited to: “HTA is currently entirely government-funded, highlighting the need for more diverse and sustainable funding sources, alongside a strong multidisciplinary workforce and active community participation in HTA processes.” | Abstract  Lines 48-49 |
| Line 83 “The research reveals…”: Would recommend rephrasing for clarity. It also appears that this is also based only on OECD data | The sentence has been revised again and it was edited to : “For instance, it is estimated that nearly 20% of total healthcare expenditure in Economic Co-operation and Development (OECD) countries is inefficiently spent” | Introduction  Lines 80-81 |
| Reference 3 cites “WHO. The world health report 2012”, but there was no World Health Report released in 2012. It is unclear what the authors are referring to. The PLOS Medicine Editors. (2012). The World Health Report 2012 That Wasn’t. PLoS Medicine, 9(9), e1001317. <https://doi.org/10.1371/journal.pmed.1001317> | The sentence cited for has been revised again and the reference was removed. | Line 117 |
| Line 116: The World Bank reference requires reformatting and appears to list Brazil’s GDP per capita as US$7,972.5 | The sentence has been edited to :” Among middle-income countries, Brazil is the largest country in Latin America, with a gross domestic product (GDP) estimated at around 7,972.5 USD per capita in 2021” |  |
| Line 310-316: Given the small numbers, I would recommend presenting these as absolute numbers | For the quantitative section, the absolute numbers has been added to all percentages for more clarity. | Results section quantitative part |
| Line 416: Rephrase “academicians” | The word has been rephrased to academics. | Line 430 |
| Line 456: Unclear what “environmental capacity” is referring to | Rephrased and it now reads: “In addition, limited information was available on the broader enabling environment and capacity for HTA, including institutional infrastructure, governance arrangements, human resources, and data systems that support HTA processes.” | Resources & Capacity section  Lines 468-470 |
| Line 513: It’s not clear to me that the authors can conclude a “high level of understanding and recognition” of HTA in Brazil. It is likely true, but the survey was not set up to evaluate a degree of understanding, instead relying on self-reported understanding. At best, it would appear that authors can refer to respondent’s self-reported levels of understanding. | The sentence was edited and it now reads as follows:  “The findings indicate a high level of understanding and recognition of the importance of HTA among Brazilian organizations across the public, private, and academic sectors, as reflected in stakeholders’ self-reported perceptions from interviews and surveys.” | Discussion section  Line 529 |
| Line 558: It might help to interrogate what “sustainable funding” for HTA actually is. Is this sufficient public funding for CONITEC, or enough private and public funding for all organizations that want to conduct HTA in Brazil? | This sentence has been re-explained, and it now reads as follows:  “The majority of respondents agreed that HTA benefits from sustainable funding, which they defined primarily as stable public financing allocated to CONITEC through government budgets. Most indicated that HTA activities are entirely or mainly government-funded, while one organization reported that its HTA activities rely predominantly on private funding. However, this perception of sustainability largely reflects funding adequacy for CONITEC and does not necessarily indicate sufficient or diversified funding for all organizations conducting HTA in Brazil.” | Lines 577-578 |
| Line 570: Inconsistent use or non-use of abbreviations like CONITEC, LMICs, etc. | The manuscript has been revised the abbreviation for CONITC was corrected. | Line 37 |
| Line 587: It might help to refer to literature on persistent challenges in judicial appeals to access health technologies not recommended by CONITEC through right-to-health litigation | More information has been added about persistent challenges related to the Judicialization of health. | Lines 600-601 |
| Figure 1: It’s unclear if this figure refers to author’s classification of institutions that report receiving HTA reports, or respondents’ perception of who receives HTA reports. This distinction, as well as the denominator for the percentages are important to clarify. | Thank you for this comment. We have clarified that Figure 1 reflects respondents’ perceptions of HTA report recipients rather than an author-defined classification. The figure caption and text now specify that percentages are calculated based on the total number of respondents to the question, with multiple responses permitted. | Results Section Fig 1 |
| Figures 2 and 3. It took me some time to understand these figures before realizing they are describing sample characteristics. The issue with the current figures is that they appear to portray a systematic classification of all professional contributions to HTA stages and outcomes, rather than self-reporting of survey respondents’ contributions. I would recommend reformatting these figures into a descriptive table to avoid this misunderstanding. | Thank you for this comment. Figures 2 and 3 present data exactly as reported by survey respondents and reflect organizations’ self-reported contributions to HTA stages and outcomes. They do not represent an author-defined or systematic classification of professional roles. We opted to retain these figures to preserve the original structure and reporting of the survey data and to ensure fidelity to respondents’ inputs. The figures are intended to be descriptive of respondents’ perspectives rather than normative representations of HTA processes. |  |
| Figure 6. What is the difference between “technical” and “guideline”? It isn’t possible to interpret the figure until this is clarified. | Description has been added to the results, and it now reads as follows: “Guidelines indicate the availability of high-level formal and normative guidance that sets expectations and standards by topic area, while technical practices reflect the operational and methodological activities used to carry out HTA.” | Fig 6 |
| References often re-order organization names in first and last name format for individuals. | The reference list has been revised and the ones related to organizations were edited | Reference List |
